# Animal Models of Pulmonary Hypertension: Matching Disease Mechanisms to Etiology of the Human Disease

**DOI:** 10.4172/2161-105X.1000198

**Published:** 2014-08-04

**Authors:** Kelley L. Colvin, Michael E. Yeager

**Affiliations:** 1 Deptartment of Pediatrics-Critical Care, University of Colorado Denver, USA; 2 Cardiovascular Pulmonary Research, USA; 3 Department of Bioengineering, University of Colorado Denver, USA; 4 Linda Crnic Institute for Down Syndrome, USA

**Keywords:** Pulmonary hypertension, Right heart failure, Therapy, Animal model

## Abstract

Recently a great deal of progress has been made in our understanding of pulmonary hypertension (PH). Research from the past 30 years has resulted in newer treatments that provide symptomatic improvements and delayed disease progression. Unfortunately, the cure for patients with this lethal syndrome remains stubbornly elusive. With the relative explosion of scientific literature regarding PH, confusion has arisen regarding animal models of the disease and their correlation to the human condition. This short review uniquely focuses on the clear and present need to better correlate mechanistic insights from existing and emerging animal models of PH to specific etiologies and histopathologies of human PH. A better understanding of the pathologic processes in various animal models and how they relate to the human disease should accelerate the development of newer and more efficacious therapies.

## Introduction

Pulmonary hypertension (PH) refers to a mean pulmonary artery pressure at rest of greater than or equal to 25 mm Hg or greater than 30 mm Hg with exercise [1]. PH may arise as an idiopathic disorder, or more commonly, associated with secondary causes [2] and is a major worldwide health burden [3]. Sadly, there remains no cure despite major advances in our understanding of the pathogenesis of PH with respect to genetics [4], vasoreactivity [5], inflammation [6], and cell and molecular biology [7].

On a more positive note, the last 30 years of research in PH has been nothing less than stunning in its breadth and depth of insight. This is evidenced by a dramatic increase in survival time and quality of life for patients with PH [8], brought about largely by incorporation of vasodilatory therapies developed from the research [5]. These advances have ushered in a “renaissance period’ of biomedical research aimed at understanding and better treating PH. As a testament to this, a PubMed search using date restrictions and with the search terms “pulmonary hypertension therapy” shows that in 1979 there were 11 published reviews, and only 6 in 1980. In 2013, there were 247! While this is certainly dramatic quantitatively it may be too early to assess some of the qualitative aspects of such a spectacular increase in the scientific literature specific to PH. The enormity of the literature regarding PH and the proliferation of new journals publishing heart lung biomedical science are both potentially contributing to difficulties moving the field ahead towards the goal of better therapies. With this review we attempt to highlight some of these problems and to bring into sharper focus potential correlations between specific preclinical models to specific forms of human PH. Finally, we humbly submit process-based recommendations to our “PH community” of clinicians, basic and translational researchers, biotech and biopharma colleagues, with the goal of increasing the efficiency of our collective efforts to find a cure.

## PH and the WHO Classification System

In 2008, the 4th World Symposium on Pulmonary Hypertension meeting at Dana Point, California [9] produced an updated clinical classification system for PH [10]. This system was based on earlier world symposia on PH in 1998 and 2003 [11]. As can be seen from Table 1, PH represents a very broad spectrum of disease etiology and pathobiology affecting not only the lungs and right ventricle directly, but also secondarily through other organ pathologies. Group I is pulmonary artery hypertension (PAH), Group II is PH associated with left heart disease, Group III is PH associated with lung disease and/or hypoxia, Group IV is PH associated with chronic thromboembolic disease, and Group 5 is PH associated with multifactorial mechanisms. Even within a single classification group, there are distinct mechanistic programs that contribute to PH, either on the arterial or venous side of the pulmonary circulation. It is obvious that no single preclinical model could be generated that would serve as an excellent surrogate to study the pathogenesis of PH, with the possible exception of high altitude PH modeled by chronic exposure to hypobaric hypoxia. It seems logical that to push forward the development of our understanding of human PH and to develop better therapies, more animal models need to be developed that recapitulate specific pathologies in ways that mirror the classification system for human PH.

## Preclinical Models: Correlations to the Classification System

As can be seen, PH is not so much a specific disease entity as it is a syndrome that may result from a number of disparate pathobiological states. Although in general, no one animal model of PH recapitulates all aspects of the severe pathology of human disease [12], these experimental models support the idea that PH is elicited by variable stimuli and that the structural changes observed in the pulmonary vasculature are related to the type of inciting event [13]. The chief histological features of pulmonary hypertensive vascular disease in humans and in animal models have been summarized elsewhere [12]. The following review of preclinical models is by no means exhaustive, but is instead meant to familiarize the reader with the main themes in the models. In mice, the classic model of chronic hypoxia produces PH with some variability across strains [14]. The development of PH is associated with varying degrees of mild to moderate muscularization of small pulmonary arterioles, likely due to hypertrophy and/or mild hyperplasia of smooth muscle cells (SMC). Adventitial thickening and infiltration by inflammatory cells into the adventitia are noted. These hypoxia-induced vascular changes are indeed mild and much less robust than in rat hypoxic PH models and reflect gene expression level differences [15]. Newer models in mice have begun to exploit the species’ capacity for genetic manipulation. For example, mice that are mutant for bone morphogenetic protein receptor type 2 (BMPR2), the most common gene mutated in human PAH to date [16], develop a mild PH in hypoxia [17]. Conditional deletion of endothelial cell BMPR2, while avoiding embryonic lethality, resulted in varying RV hypertrophy and vascular remodeling [18], implying that BMPR2 loss is neither necessary nor sufficient for PH and RV failure [14]. Other genetically engineered mice have been generated that examine immune dysfunction. Mice that over-express interleukin- 6 (IL-6) develop severe PH, neointimal proliferation, and RV hypertrophy upon chronic hypoxia exposure [19]. In humans with systemic sclerosis, PAH is one of the leading causes of death [20].

In mice transgenic for Fra-2 (Fos-related antigen-2, a Jun/Fos transcription factor family member), increases in metalloprotease activity and PASMC proliferation are exaggerated and contribute to pulmonary vascular remodeling [21]. Mice with mutations in vasoactive genes are also models of PH, as is the case for the moderate PH and RV hypertrophy observed in mice lacking vasointestinal peptide [22]. It should be noted that the gender differences in the incidence of human IPAH [3], are observed in at least one preclinical model used for the study of PH. For example, in mice overexpressing S100A4/MTS-1/FSP-1, increased right ventricular systolic pressure is observed [23]. In approximately 5% of these mice, pulmonary vascular remodeling and the development of plexiform-like lesions occurs, almost exclusively in females [24]. Although beyond the scope of this review, the reader is directed towards excellent reviews on the subject of gender differences and PH [25,26].

Mouse models have begun to explore the role of the immune system in the pathogenesis of PH. Schistosomiasis-associated is one of the most common causes of PAH worldwide, with an estimated ~ 200 million infected persons of which about 1% develop the disease [27]. In mice with schistosomiasis-PAH, granulomatous lesions and pulmonary vascular remodeling is heterogeneously localized in the lung [28], and appears to require transforming growth factor beta and IL-4/IL-13 signaled myelomonocytic cells [29]. Fulminant Th2 inflammatory responses and vascular remodeling in the lung have also been examined in mice sensitized with ovalbumin and further challenged in the airways [30] and with ovalbumin or Aspergillus antigen challenge in the peritoneal cavity [31]. Much of the histopathology in these models seems to be most prevalent in the more proximal airways and pulmonary vessels, in addition to changes at the level of resistance vessels. Intriguingly, the Th2 response determines the extent of pulmonary vascular remodeling, is sufficient to cause the lung remodeling, and may share common disease pathways and mediators with chronic hypoxia PH [32].

An excellent summary table of mouse models from 1996–2011 and their respective pathologies is available [14]. Rats have been used for decades to study PH, particularly the chronic hypoxia and monocrotaline (MCT) models. Exposure of rats to hypobaric hypoxia causes a doubling of mean pulmonary artery pressure, progressive structural change in the pulmonary vasculature, is attended by influx of inflammatory cells (predominantly myeloid lineage), but RV failure is absent [3]. Strain differences are well appreciated, with the prime example of the fawn-hooded rat, which develops more severe PH and remodeling than most other strains when exposed to hypoxia [33]. MCT is a toxic alkaloid that has been used to induce experimental PAH for decades [34]. MCT causes a widespread pneumotoxicity [35] that is reflected as a increased index of cell proliferation, which varies in time and space among the vascular and airway structures, from alveolar regions to larger bronchovascular structures [36]. Sugen 5416 vascular endothelial growth factor receptor 2 (VEGFR2) blockade, in combination with hypobaric hypoxia (Su-Hx), induces a severe PH with elements of inflammation and angio-obliteration culminating in RV failure [37]. The underlying mechanism is thought to involve primarily pulmonary artery endothelial cell death followed by the emergence of an apoptosis-resistant cell [38]. Interestingly, Su-Hx rats returned to normoxia (Su-Hx-Nx) for 10–11 weeks develop neointimal lesions closely resembling human plexogenic arteriopathy [39]. The possibility that these neointimal lesions may be reversible [13,40], that they do not fully recapitulate human plexiform lesions [13], and the lack of death from RV failure development among some research groups (for example, “a few out of ~ 300 rats” [41]), warrants further detailed investigations. This report clearly demonstrates that the plexiform lesions develop in this rat model at very late stages, leading the authors to conclude that sustained exposure to high blood pressure may be the major factor required for their development [41]. This raises the possibility that the emergence of apoptosis-resistant and hyperproliferative EC by SU5416 is the result of exposure to high shear stress secondary to the pulmonary arterial remodeling [38]. Rat strain susceptibility, as in hypoxia alone, may explain differences in the incidence of RV failure in this model.

Newer rat models of PH have recently been published. As in mice, Th2 mediated inflammation leads to pulmonary vascular remodeling that can be quite severe. Sugen 5416 blockade in combination with ovalbumin sensitization produced a robust angio-proliferative PAH that was preventable by 1) caspase inhibition, 2) dexamethasone, and 3) B lymphocyte depletion [42]. In contrast to the Th2 mouse models described above, Th2 inflammation and remodeling in the rat lung is associated with severe PAH [42]. Recently, a rat model of moderate PAH (~40 mm Hg or greater) was generated by passive transfer of autoantibodies purified from the plasma of MCT rats [43]. This was associated with pulmonary vascular remodeling, including intimal occlusion but without plexogenic arteriopathy. Intriguingly, bronchus-associated lymphoid tissues appeared to play a major role in the generation of autoantibodies [43]. Collectively, these models promote the idea that both the airways and the immune system are potentially important players in pulmonary vascular remodeling, thus deserving further study. With regard to the airways, a recent review summarized the current experimental and clinical findings that establish linkage between airway responses (ex: antigens, pollution) and the lung vasculature [44]. In any case, the possibility that the airways contribute somehow to certain forms of PH is beginning to be robustly explored.

Ungulates, such as cows and sheep, have served as excellent models for PH. Seminal studies of the effects of high altitude in these animals conducted several decades ago established many of the fundamental tenets of cardiopulmonary biology [45]. Generally speaking, cattle exposed to hypoxia develop severe PH, while sheep do not [45]. Neonatal calves are particularly sensitive to chronic hypobaric hypoxia, and exhibit remarkable intimal thickening of the media and adventitia of pulmonary vessels with mononuclear cell infiltration. As is the case for rodents exposed to chronic hypoxia, these changes are reversible [3]. Gene expression and single nucleotide polymorphism studies in cows may provide clues as to genetic predisposition to high altitude PH [46]. Along with their many positive attributes as models of PH, large animals are uniquely challenging in many respects. For example, the performance of cell lineage tracing studies, or the testing of potential therapies are difficult given that large size and cost are prohibitive for many researchers. Overcoming such obstacles may be of great benefit to the understanding of the pathogenesis of PH.

A great deal of literature is available focusing on pulmonary artery hypertension, sometimes called ascites syndrome, in broiler chickens [47]. The broiler chicken model has been investigated for decades with regard to nearly every aspect of the pathobiology of human PH: vasoactive mediators [48], genetic susceptibilities [49], microparticles [50], plexogenic arteriopathy [51], right heart failure [52], inflammation [53], lipopolysaccharide exposures [54], myeloid cell biology [55], and serotonin [56]. One of the reasons why broiler chickens are so sensitive to the development of PH is the rate of rapid growth from chicks to adults. In an 8-week span, a 40 g chick develops into a 4,000g broiler, the equivalent of a 3 kg human newborn baby weighing 300 kg after 2 months [47]. Several investigations (summarized in [47]) point to the lack of pulmonary vascular capacity in chickens compared to humans. Despite extensive investigations into these areas and findings in the model that are very close to human PAH, this literature is predominantly cited by those in poultry sciences and almost never cited by other investigators (M. Yeager, unpublished Web of Science Citation Index v.5.13.2 search, April 23, 2014, search terms: pulmonary, hypertension, chicken).

As can be seen in Table 1, PAH can be associated with human immunodeficiency virus infection (HIV) (Group 1.4.2). The incidence of PAH is much higher in HIV-infected individuals, and its pathogenesis has been recently reviewed [57]. Macaque monkeys infected with SHIV-nef (a chimeric viral construct containing the HIV nef gene in a simian immunodeficiency virus [SIV] backbone) develop complex plexiform–like lesions [58]. These lesions appear very similar to those found in patients with HIVPAH [59]. Whether the pulmonary vascular remodeling in the SHIV-nef model is associated with PAH and/or contributes to RV failure is unknown, but evidence for RV hypertrophy was absent [58].

Several animal models are available to study congenital abnormalities and pulmonary hypertension in children. Congenital diaphragmatic hernia (CDH) occurs in about 1 in 3,000 births and is associated with pulmonary hypoplasia and persistent pulmonary hypertension [60]. Surgical approaches [61], pharmacologic approaches [62], and genetic models [60] are in use to study the development of CDH-PAH. The lung histopathology in preterm baboon and preterm lamb models appears to closely recapitulate the histopathological appearance of preterm infants with bronchopulmonary dysplasia (BPD) [63]. Congenital heart diseases in which pulmonary blood flow increases commonly lead to the development of pulmonary hypertension [64]. An excellent model for this has been developed by Fineman et al. in which an aortopulmonary shunt is created in late gestation fetal lambs [65]. By 1 month of life, shunted lambs reproduce the salient features of the human disease, namely postnatal pulmonary hypertension, increased pulmonary blood flow, and vascular remodeling [65]. The mechanisms that impel the disease in the lambs is largely attributed to reactive oxygen and reactive nitrogen species that drive the dysregulation of key signaling pathways in pulmonary artery endothelial and smooth muscle cells that leads to remodeling and increased pulmonary artery pressure [64]. Similar results have been published using a piglet model of PAH with right heart failure caused by prolonged overcirculation via an anastomosis between the left innominate artery and the pulmonary arterial trunk [66]. In this model, the development of PAH is speculated to be propelled by dysfunction of voltage-gated potassium channels, angiogenic factors, and inflammation [66,67]. Chronic thromboembolic pulmonary hypertension (CTEPH) is caused by persistent organized clots in the pulmonary arterial tree and results in distal pulmonary vasculopathy [68]. The disease is thought to involve two compartments in the lung, the obstructed vascular bed and those unobstructed beds presumably exposed to higher fluid forces [68]. CTEPH is difficult to model since thrombi are notoriously fleeting due to the robust fibrinolytic capacity of the pulmonary vasculature [69]. The distal pulmonary vasculopathy found in CTEPH is similar to that seen in IPAH [70]. Recently, an excellent review of rat, mouse, pig, and primate models for CTEPH, with their strengths and weaknesses has been published [71].

To summarize, all preclinical models of PH share some common pathologies while also displaying unique differences. In Table 2, we remind the reader of the current histopathological classification system, which has continually developed out of the pioneering work of Wagenvoort [72,73] and Heath and Edwards [74]. In Table 3 we have attempted to correlate these pathologies to those of human PH and within the current classification system. Some models, such as the MCT rat, defy simple categorization because of multiple organ pathology, and/or complex and mixed histopathology.

## Matching Rodent Models of PH to Human PH

Several recently published reviews regarding PH summarize novel and emerging therapies [75,76], pathology [13], clinical trial designs [77], and cell type-specific contributions to the disease process [78]. As a thought experiment in therapy development for PH, in the following section we compare and contrast the two most common animal models of PH. Currently, the aim of the majority of therapies for PH is increased pulmonary vasodilatation [75]. Many of the emerging therapies target inflammation and intracellular signaling pathways potentially operational in PAEC, PASMC and pulmonary artery fibroblasts [78]. In the past ~10 years, a hypothesis of pulmonary artery endothelial cell death and subsequent resistance to apoptosis and exuberant proliferation has been favored [79]. As such, the focus on PAEC and PASMC has only intensified. This hypothesis, which one of us has helped in small ways to push forward [80], is based on studies in human lung tissue from patients with PAH focusing on the plexiform lesion and in occlusive neointimal lesions more generally [81]. These data appear to be substantiated by studies in the MCT and Sugen-hypoxia rat models in which PAEC and PASMC proliferation have been noted [3]. However, in the MCT model there is widespread pneumotoxicity [82], including airway and alveolar dysfunction [83,84], a feature also noted in human PAH [85,86]. To date, very few studies, if any, have explored the possibility that early bronchial epithelial cell death could contribute to the pathobiology in this model. Indeed, there may be substantial cell death at the level of the bronchus as well as the alveolus that occurs within 1–2 days after MCT administration (M. Yeager, unpublished observations). As a side note, the contribution of the hepatosplenomegaly seen in MCT rats, despite its nearly universal finding across studies, has never been mechanistically explored either. PAEC and PASMC apoptosis is of course observed, which led to the hypothesis that these cell types are likely the principal players in the disease process. However, it cannot be overstated that the MCT model represents a complex multi-organ disease process that culminates in PAH and RV failure [3]. The fact that so many therapeutic approaches appear to prevent and even reverse MCT PAH has been used to disdainfully criticize the model as a poor approximate for human PAH. A closer examination of these data reveals that most of the studies sacrificed the rats at ~ 4–5 weeks post MCT administration to reveal a lower grade of cardiopulmonary pathology (but still on a spectrum of pathology) and thus declared essentially as “cures”. Future studies should allow for MCT rats (or any animal model of PH) that are treated therapeutically to live out longer to test the true durability of the approach. Finally, it must be acknowledged that apoptosis of cells other than PAEC and PASMC may be critically important to the appropriate interpretation of data. For example, if an apoptosis inhibitor is used to reverse MCT PH, is the effect primarily upon PAEC and PASMC, or might other affected cell types be as or more important? These cells and the molecular pathological underpinnings may represent an untapped wealth of potential therapeutic targets.

The Sugen-hypoxia model is an important preclinical model of PAH [37], with many of its most salient features having been replicated repeatedly by numerous research groups. Several studies and reviews conclude that the pathological lesions present in the distal vasculature of the Su-Hx rats are “indistinguishable” from those in human PAH (summarized in [3]). However, recent reports directly assessing the pathology conclude that even the Su-Hx model lesions do not fully recapitulate plexogenic lesions in humans [13]. In summary, the findings that 1) in humans there is a spectrum of pathological change in the pulmonary vasculature even in “normal control” lungs, 2) ~ 70% of human PAH is associated with occlusive neointimal lesions [87,88] and are therefore not a requirement for PAH, 3) severity of PAH in the Su-Hx model does not directly correlate with the extent of occlusive lesions [13], and 4) the Su-Hx rat model may be reversible upon return to normoxia [13], reinforce the notion that the Su-Hx model, as is the case for any preclinical model, is best suited to study some forms of PAH and not ideally suited for others (as has been suggested previously [89]). Along a similar line of argument, as MCT rats do not recapitulate plexiform-like lesions, it may be unwise to study them for that purpose. However, the changes observed in the more proximal bronchovascular structures of MCT rats, including formation of active tertiary lymphoid tissues [43], very closely replicates some forms of human PH [90]. Recently, it was reported that idiopathic PAH recurred in a patient approximately 12 months following lung transplantation (Narula et al., CHEST, 2014; 145(3_Meeting Abstracts:624A). This finding, and others like it following lung transplants in IPAH (Muzaffar et al., CHEST, 2008; 134(4_Meeting Abstracts:c16002) and chronic granulomatous lung disease [91], if confirmed by larger, published studies, would call into question the assumption that the lung itself is the principal source of the disease process. Considering the medical axiom “ablata causa tollitur effectus” (if the cause is taken away, its effect will disappear), the assumption that there must be a lung-specific origin of the disease may be inappropriate, at least in some cases. Although the data are very limited, these studies point to the possibility that in IPAH, where there is no other obvious ongoing disease process, the phenomena of cell proliferation, apoptosis resistance, and pulmonary vascular remodeling may not be central drivers of the disease but are manifestations of a disease originating outside of the lung that somehow preferentially targets the lung and possibly the right ventricle.

## Specific Recommendations

We share the collective goal of delivering improved therapies, preferably custom tailored to a PH patient’s particular apparent etiology. To accomplish this, it is our belief that several key changes (numbered in parentheses that follows) need to occur within the PH biomedical community.

(1) We assert that there is a need to increase the communication between basic scientists and clinicians in the workshops and symposia involved in the development and testing of new therapies for PH. Such contributions would hopefully lead to a more pervasive and comprehensive understanding of the latitude of lung, RV, and other organ injuries in the preclinical models.
(2) To do this, it is imperative for us to (more) closely document, compare, and contrast the pathobiological findings of the preclinical models. Doing so will more dramatically reveal similarities and differences in PH across the models in ways that mirror the differences observed in human PH.
(3) This will require research groups to adhere to the highest ethical standards of research practice, and more fully disclose the number of animals in experimental groups, the extent to which the investigators looked at specific effects (proliferation, apoptosis, etc.) in the lung and RV, and perhaps other organ systems, and the consistency of the findings.

Research findings submitted for publication and/or presented at conferences should be constructively evaluated by the PH scientific community, with an eye towards

(4) Placing the experimental results in the context of the preclinical models used vis-à-vis human PH. As a research community, of course we need to work towards new avenues of research but we should never discount the value of
(5) Validating (or not validating) other groups’ findings. Such efforts, whether they are in agreement or disagreement with other studies, should be welcomed for publication. Going forward, our goal of developing novel, personalized, and more efficacious therapies will be expedited by
(6) Presenting data with a clear rationale for the preclinical model chosen (as has been suggested previously [89]), and framing the interpretation of the main findings in as accurate and complete historical context as possible.

With the advent of so many new journals, as well as the huge increase in the number of PH publications per year, the production and dissemination of high quality research will likely prove a formidable challenge.

## Figures and Tables

**Table 1:** Current Classification for Pulmonary Hypertension [2]

1. Pulmonary Arterial Hypertension (PAH)
1.1. Idiopathic (IPAH)
1.2. Heritable/familial (FPAH) 1.2.1. BMPR2
1.2.2. ALK1, Endoglin
1.2.3. Unknown
1.3. Drug and toxin-induced
1.4. Associated with (APAH)
1.4.1. Conective tissue disorders
1.4.2. HIV infection
1.4.3. Portal hypertension
1.4.4. Congenital heart diseases
1.4.5. Schistosomiasis
1.4.6. Chronic hemolytic anemia
1.5. Persistent pulmonary hypertension of the newborn (PPHN)
1′. Pulmonary veno-occlusive disease (PVOD) and pulmonary capillary hemangiomatosis (PCH)
2. Pulmonary hypertension with left heart disease
2.1. Systolic dysfunction
2.2. Diastolic dysfunction
2.3. Valvular disease
3. Pulmonary hypertension due to lung diseases and/or hypoxia
3.1. Chronic obstructive pulmonary disease (COPD)
3.2. Interstitial lung disease
3.3. Other pulmonary diseases with mixed restrictive and obstructive pattern
3.4. Sleep disordered breathing
3.5. Alveolar hyperventilation disorders
3.6. Chronic exposure of high altitude
3.7. Developmental abnormalities
4. Chronic thromboembolic pulmonary hypertension (CTEPH)
5. Pulmonary hypertension with indistinct, multi-factorial mechanisms
5.1. Hematological disorders (e.g. myeloproliferative disorders, splenectomy, hemoglobinopathies)
5.2. Systemic disorders (e.g. sarcoidosis, pulmonary Langerhans cell histocytosis, lymphangiomatosis)
5.3. Metabolic disorders (e.g. glycogen storage disease, Gaucher’s disease, thyroid disorders)
5.4. Others (e.g. tumoral obstruction, fibrosing mediastinitis, chronic renal failure and dialysis)

**Table 2:** Major Histopathological Features of Pulmonary Hypertensive Vascular Disease by Dana Point 2008 Clinical Classification [3]

Group 1: PAH
1.1–1.4. Pulmonary plexogenic arteriopathy
Early phase:
• Medical hypertrophy
• Cellular intimal proliferation of muscular pulmonary arteries
• Appearance of muscle in normally nonmuscular arteries
Late phase:
• Concentric laminar intimal fibrosis
• Loss of luminal vascular volume
• Dilatation lesions (vein-like branches, angiomatoid lesions)
• Plexiform lesions
• Recanalization of arteries
• Fibrinoid necrosis
• Arteritis
Group 1′: PVOD
• Foci of intense congestion of pulmonary parenchyma
• Patchy hemosiderosis associated with areas of congestion
• Encrustation of elastin with iron and calcium salts in congested areas
• Duplication of elastic laminae
• Obliterative fibrosis of small veins and of venules, associated with congested areas
• Abnormalities set against a background of normal or near normal lung tissue
• Prominence of capillaries, associated with increased numbers of capillaries, often blurring the distinction from pulmonary capillary hemangiomatosis (group 1.4.2)
Group 1′: PCH
• Marked increase and prominence of capillary vessels in alveolar walls, interlobular septa, bronchovascular bundles, and pleura; masses of capillaries may bulge into lumina of airways and vessels
• Associated features of PVOD in some cases
Group 2: Pulmonary hypertension with left heart disease
• Arterialization of large or middle-sized pulmonary veins
• Interstitial edema and fibrosis
• Hemosiderosis
• Medial hypertrophy and adventitial thickening of pulmonary arteries
Group 3: Pulmonary hypertension associated with lung disease and/or hypoxemia
3.1 and 3.3–3.5. Hypoxic pulmonary vasculopathy
• Intimal proliferation; adventitial thickening
• Medial hypertrophy of muscular pulmonary arteries and arterioles, especially of smaller branches
• Longitudinally oriented intimal smooth muscle cells
• Slight medial hypertrophy of veins
3.2. Pulmonary vasculopathy associated with interstitial lung disease
• Features of hypoxic pulmonary vasculopathy
• Eccentric intimal fibrosis of arteries and, to a lesser extent, veins
Group 4: Pulmonary hypertension due to chronic thrombotic and/or embolic disease
• Thromboembolic obstruction of distal pulmonary arteries
Eccentric intimal fibrosis
Recanalized organized thrombi forming bands and webs
Fresh thrombi very rare
Nota bene: lesion may be focal, requiring extensive search in multiple sections
• Nonthrombotic pulmonary embolism
Nonthrombotic material or tissue (foreign bodies, bone marrow)
Fat embolism: many dilated optically empty blood vessels (down to capillary size)
Group 5: Miscellaneous [sarcoidosis, compression of pulmonary vessels (adenopathy), tumor, fibrosing mediastinitis]
• Heterogeneous group of disorders, some showing the features of congestive vasculopathy, some with features of post-thrombotic vasculopathy, some with combinations

**Table 3:** Correlation of Histopathological Features of Human Pulmonary Hypertensive Vascular Disease to Preclinical Models Human Animal Model

Human	Animal Model
Group 1: PAH	
Early phase: Medical hypertrophy Cellular intimal proliferation of muscular pulmonary arteries Appearance of muscle in normally nonmuscular arteries	OVA/Asp challenge mice; Su/OVA rat BMPR2 mutant mice; VIP−/− mouse Su-Hx; CDH/PPHN baboon/lamb MCT rat; Autoab transfer rat S100A4/MTS-1 over expressing mouse Fra2 Tg mouse; IL-6 over expressing mouse Schisto mouse; Neprilysin null mouse
Late phase: Concentric laminar intimal fibrosis Loss of luminal vascular volume Dilatation lesions (vein-like branches, angiomatoid lesions) Plexiform lesions Recanalization of arteries Fibrinoid necrosis Arteritis	Broiler chicken Su-Hx-Normoxia S100A4/MTS-1 over expressing mouse MCT pneumonectomy rat SHIV-nef
Group 1′: PVOD Foci of intense congestion of pulmonary parenchyma Patchy hemosiderosis associated with areas of congestion Encrustation of elastin with iron and calcium salts Duplication of elastic laminae Obliterative fibrosis of small veins/venules Abnormalities set against a background of near normal lung Prominence of capillaries	MCT? Su-Hx?
Group 1′: PCH	
Marked increase/prominence of capillary vessels in alveoli interlobular septa, bronchovascular bundles, and pleura; masses of capillaries may bulge into lumina of airways and vessels Associated features of PVOD in some cases	
Group 2: Pulmonary hypertension with left heart disease Arterialization of large or middle-sized pulmonary veins Interstitial edema and fibrosis Hemosiderosis Medial hypertrophy/adventitial thickening of pulmonary arteries	
Group 3: Pulmonary hypertension associated with lung disease and/or hypoxemia 3.1 and 3.3–3.5. Hypoxic pulmonary vasculopathy Muscularization of arterioles Medial hypertrophy of muscular pulmonary arteries Longitudinally oriented intimal smooth muscle cells Slight medial hypertrophy of veins 3.2. Pulmonary vasculopathy associated with interstitial lung disease Features of hypoxic pulmonary vasculopathy Eccentric intimal fibrosis of arteries and, to a lesser extent, veins	Broiler chicken Chronic hypoxia-Neonatal calf Su-Hx rat Fawn Hooded rat Chronic hypoxia-mouse Chronic hypoxia-rat Chronic hypoxia + MCT rat
Group 4: Pulmonary hypertension due to chronic thrombotic/embolic disease Thromboembolic obstruction of distal pulmonary arteries Eccentric intimal fibrosis Recanalized organized thrombi forming bands and webs Fresh thrombi very rare Nonthrombotic pulmonary embolism Nonthrombotic material (foreign body/bone marrow) Fat embolism	Vena cava ligation + thrombi-pig, rat, primate Vena cava ligation + stenosis-mouse, rat, De-endothelialization + 50–80% jugular vein stenosis-pig
Group 5: Miscellaneous [sarcoidosis, compression of pulmonary vessels (adenopathy), tumor, fibrosing mediastinitis]	
Heterogeneous group of disorders, some showing features of congestive vasculopathy, some postthrombotic vasculopathy	
